# Erythropoietin Exacerbates Inflammation and Increases the Mortality of *Histoplasma capsulatum*-Infected Mice

**DOI:** 10.1155/2015/786319

**Published:** 2015-10-11

**Authors:** Gisele Aparecida Locachevic, Priscilla Aparecida Tartari Pereira, Adriana Secatto, Caroline Fontanari, Alyne Fávero Galvão, Morgana Kelly Borges Prado, Karina Furlani Zoccal, Tânia Petta, Luiz Alberto Beraldo Moraes, Simone Gusmão Ramos, Fabíola Attie de Castro, Carlos Artério Sorgi, Lúcia Helena Faccioli

**Affiliations:** ^1^Departamento de Análises Clínicas, Toxicológicas e Bromatológicas, Faculdade de Ciências Farmacêuticas de Ribeirão Preto, Universidade de São Paulo, 14040-903 Ribeirão Preto, SP, Brazil; ^2^Departamento de Química, Faculdade de Filosofia, Ciências e Letras de Ribeirão Preto, Universidade de São Paulo, 14040-901 Ribeirão Preto, SP, Brazil; ^3^Departamento de Patologia e Medicina Legal, Faculdade de Medicina de Ribeirão Preto, Universidade de São Paulo, 14049-900 Ribeirão Preto, SP, Brazil

## Abstract

Erythropoietin (EPO) is a key hormone involved in red blood cell formation, but its effects on nonerythroid cells, such as macrophages, have not been described. Macrophages are key cells in controlling histoplasmosis, a fungal infection caused by *Histoplasma capsulatum* (*Hc*). Considering that little is known about EPO's role during fungal infections and its capacity to activate macrophages, in this study we investigated the impact of EPO pretreatment on the alveolar immune response during *Hc* infection. The consequence of EPO pretreatment on fungal infection was determined by evaluating animal survival, fungal burden, activation of bronchoalveolar macrophages, inflammatory mediator release, and lung inflammation. Pretreatment with EPO diminished mononuclear cell numbers, increased the recruitment of F4/80^+^/CD80^+^ and F4/80^+^/CD86^+^ cells to the bronchoalveolar space, induced higher production of IFN-*γ*, IL-6, MIP-1*α*, MCP-1, and LTB_4_, reduced PGE_2_ concentration, and did not affect fungal burden. As a consequence, we observed an increase in lung inflammation with extensive tissue damage that might account for augmented mouse mortality after infection. Our results demonstrate for the first time that EPO treatment has a deleterious impact on lung immune responses during fungal infection.

## 1. Introduction

Histoplasmosis caused by the dimorphic fungus* Histoplasma capsulatum* (*Hc*) is a granulomatous disease that primarily affects the lungs [1]; however, depending on the immune status of the host and the inhaled fungal load, the infection can progress to chronic pulmonary and disseminated diseases leading to death [2]. Alveolar macrophages are the main cell type involved in the phagocytosis and death of* Hc*. These yeasts may multiply inside macrophages, inducing cell death. However, when an appropriate immune response is triggered, alveolar macrophages are activated, killing intracellular* Hc* and controlling the infection [3]. Host control and eradication of* Hc* infection are mediated by a T helper type 1- (Th1-) mediated cellular immune response, which is associated with production of IL-12, IFN-*γ*, and TNF-*α*, and activation of macrophages, dendritic cells, and T helper lymphocytes [1]. Besides cytokines, lipid mediators are essential for host defense against* Hc*. Previously, we demonstrated that leukotrienes (LTs), lipid mediators derived from arachidonic acid metabolism [4], play an important role in primary and secondary immune responses during histoplasmosis by increasing phagocytosis and killing by macrophages [5–7]. By contrast, prostaglandins (PGs) enhance* Hc* infection [8]. Innate, sentinel dendritic cells, neutrophils, and mainly tissue macrophages are major producers of eicosanoids [9], as well as cytokines [10].

Considering the importance of macrophages for infection control, we can suggest that the enhancement of their effector activities might be critical and decisive to the fate of infections. We found a study by Lifshitz et al. [11] that shows that treatment with erythropoietin (EPO) activates the biological functions of macrophages. They observed that peritoneal macrophages and bone marrow-derived macrophages from EPO-treated mice have increased phagocytic rates and nitric oxide production but a reduction in IL-12 and IL-10. Indeed, macrophages are now considered a target for EPO.

EPO is a hormone that is classically known for regulating red blood cell production. In adults, EPO is produced and secreted primarily by kidney tubular cells [12, 13]. The nonerythroid effects of EPO have been described [14, 15] such as their role in immune system activation for controlling some infections. Nishimura et al. [16] showed that the absence of EPO production decreases bone marrow erythropoiesis and aggravates anemia during* Trypanosoma brucei brucei* infection. In addition, EPO has been administered to patients with infectious diseases to treat pathogen-induced hemolytic anemia [17]. During* Plasmodium berghei* infection, administration of EPO prolonged mouse survival without affecting parasitemia. This phenomenon is likely due to the fact that EPO ameliorated parasite-induced anemia, reduced TNF-*α* and IFN-*γ* production, and decreased neuronal apoptosis, representing a potential therapeutic benefit in cerebral malaria [18]. In contrast to that reported for protozoal infections, EPO treatment in cases of* Salmonella typhimurium* infection increased the mortality of mice and decreased the production of IL-6, TNF-*α*, and IL-1*β* in the liver [19]. To the best of our knowledge, there are no reports evaluating the effect of EPO pretreatment in fungal infections such as histoplasmosis.

Investigations of the relationship between EPO and LTs and PGs have been somewhat limited. Mason-Garcia et al. [20] demonstrated that erythrocytes in the presence of EPO increase the production of LTB_4_ and 12-hydroxyeicosatetraenoic acid, and they suggested that this increase may be related to the activation of phospholipase A_2_ and C. However, nothing is known about the relationship between EPO and lipid mediator production in fungal infections. The role of EPO in PG synthesis or action has not been described, although some studies demonstrated the effects of PGs in EPO production or action [21–23]. Considering that macrophages and lipid mediators are important for the control of* Hc* infection and that EPO might act on these two parameters, our aim was to investigate the effects of EPO pretreatment on experimental histoplasmosis. We found that EPO pretreatment increased the mortality of infected mice, the number of cells expressing CD80 and CD86 in the F4/80^+^ population, and the production of inflammatory cytokines, chemokines, and LTB_4_ and upregulated late apoptosis. On the other hand, no alteration of fungal burden (CFU) was observed, and the number of mononuclear cells recruited to bronchoalveolar lavage fluid (BALF) was reduced, as well as PGE_2_ production. When extrapolated to humans, our study suggests that the use of EPO for the treatment of anemia in patients with chronic renal disease might impair the control of fungal infections such as those caused by* Hc*, leading to lung inflammation, tissue damage, and death.

## 2. Materials and Methods

### 2.1. Mice

Male, 8-week-old C57Bl/6 mice were obtained from the Faculdade de Ciências Farmacêuticas de Ribeirão Preto, Universidade de São Paulo (Ribeirão Preto, SP, Brazil). Uninfected mice, treated with EPO and untreated, were kept at the animal facility of Faculdade de Ciências Farmacêuticas de Ribeirão Preto with a 12 h light/dark cycle and with water and food* ad libitum*. After* Hc* infection, mice were kept in a biohazard facility in safety enclosures and under the same standard husbandry conditions used for uninfected mice. All procedures were performed following the guidelines of the Animal Care Committee of Universidade de São Paulo (protocol number 11.1.464.53.0).

### 2.2. EPO Treatment

Recombinant human EPO (rHuEPO; HEMAX, from Biosintética-Aché, Brazil) was diluted in phosphate-buffered saline (PBS) before use. The dose was chosen based on that used by Lifshitz et al. [11] and on a dose-response (45, 50, 100, 150, and 180 U of EPO) trial performed in our laboratory as pilot experiments (data not shown). The dose of 45 U of EPO in 100 *μ*L of PBS was selected and administered subcutaneously to each mouse three times per week for one week prior to* Hc* infection.

### 2.3. *Histoplasma capsulatum* Strain and Infection

The* Hc* isolate used in this study was obtained from a patient at the Hospital das Clínicas de Ribeirão Preto (Ribeirão Preto, SP, Brazil) and maintained in our lab. The mycelial phase of* Hc* was obtained as previously described [5]. To obtain yeasts, mycelia were cultivated at 37°C in BHI blood agar. Samples were used when fungal viability was ≥90%, based on fluorescein diacetate and ethidium bromide exclusion assays, performed as previously described [24].

Mice were infected with* Hc* as previously described [5]. Briefly, animals were anesthetized, and their tracheae were exposed. Then 100 *μ*L of PBS without or with a sublethal (5 × 10^5^ yeasts/mouse) or lethal (1 × 10^6^ yeasts/mouse) inoculum of viable* Hc* cells was inoculated intratracheally (i.t.). Mice were euthanized 14 days after infection in a CO_2_ chamber for lung, spleen, and bronchoalveolar fluid analyses. Uninfected and untreated mice were euthanized on the same day as infected animals.

### 2.4. Experimental Design

In this study, we used the following 4 experimental groups of mice to evaluate the effect of EPO pretreatment in* Hc* infection: (1) untreated and uninfected mice (*control*), (2) uninfected (but inoculated i.t. with 100 *μ*L PBS) mice treated with EPO s.c. for one week (*EPO group*), (3) untreated (but inoculated s.c. with 100 *μ*L of PBS) mice infected i.t. with* Hc* (*Hc group*), and (4) mice pretreated s.c. with EPO for one week and then infected i.t. with* Hc* (*EPO* +* Hc group*).

### 2.5. Bronchoalveolar Lavage Fluid (BALF) Collection

BALF was harvested by carefully injecting 1 mL of PBS into the lungs. This procedure was repeated three times and the recovered fluid was pooled. Total cell counts in BALF samples were achieved using a Neubauer chamber, and differential cell counts were performed using cytospin preparations stained with Panoptic (Laborclin, Paraná, Brazil) [5].

### 2.6. Assessment of Fungal Burden in the Lungs and Spleen

The recovery of* Hc* from mouse lung and spleen was performed as previously described [25]. Two serial dilutions of tissue homogenates were made in PBS and 100 *μ*L of each diluted homogenate sample was placed on BHI-agar-blood medium and then incubated for 15 days at 37°C and with 5% CO_2_. Fungal colonies were counted manually and the fungal burden was expressed as the number of colony-forming units per gram of tissue (CFU/g).

### 2.7. Histology

Lungs collected from mice 14 days after infection were fixed in 10% formalin, processed, and embedded in paraffin blocks, from which 5 *μ*m sections were cut and stained with hematoxylin and eosin (H&E). Stained tissue sections were analyzed with a Leica DMR light microscope (Leica Microsystems GmbH, Wetzlar, Germany) equipped with a video camera (Leica Microsystems Ltd., Heerbrugg, Switzerland). Images were analyzed using the Leica Application Suite software (Leica Microsystems Image Solutions, Cambridge, UK), and the lung-lesion area (LLA) was estimated by computerized planimetry analysis of the images using the CS3 Photoshop software (Adobe, San Jose, CA, USA) and expressed as a percentage of the total lung tissue area using the formula (LLA/TA) × 100, where TA is the total lung tissue area.

### 2.8. Measurement of PGE_2_ and LTB_4_ Concentrations

To obtain tissue homogenates, lungs were removed from mice immediately after euthanasia and homogenized using a Mixer Homogenizer (Labortechnik, Wasserburg am Bodensee, Germany) in 2 mL of RPMI-1640 containing 10 *μ*M indomethacin and 1 mM EDTA. After centrifugation, supernatants were recovered, filtered, and acidified to pH 3.4–3.6 using 1 N HCl and applied slowly to a Sep-Pak C_18_ column (Waters, Milford, MA, USA) previously washed with 20 mL of 35% ethanol and 20 mL of water. After washing the columns with water, the lipids were eluted with 2 mL of absolute ethanol, dried under vacuum, and reconstituted in 100 *μ*L of methanol. Samples were centrifuged to remove any indomethacin precipitate, and the supernatants were transferred to appropriate vials for determination of the concentration of PGE_2_ and LTB_4_ by high-performance liquid chromatography-tandem mass spectrometry using an Acquity UPLC-MS system coupled to a Xevo TQ-S mass spectrometer (Waters). Chromatographic separation was conducted using a Supelco Ascentis EXPRESS C_18_ HPLC column (Sigma-Aldrich, St. Louis, MO, EUA) with dimensions of 100 mm × 3.0 mm, 2.7 *μ*m. A binary gradient system was employed in which phases A and B consisted of water/acetonitrile/acetic acid (70 : 30 : 0.02, v/v) and acetonitrile/isopropanol (70 : 30, v/v), respectively. Samples (10 *μ*L) were eluted with a flow rate of 0.6 mL/min, with a linear gradient starting with 0% B, which was increased to 15% B at 2 min, 20% B at 5 min, 35% B at 8 min, 40% B at 11 min, 100% B at 15 min, 100% B at 18 min, and 0% B at 19 min and held there for 30 min. Analyses were performed using multiple reaction monitoring (MRM) scan mode employing negative ionization. MRM transitions were 351.2 → 171 for PGE_2_ and 335.1 → 195 for LTB_4_. Quantification was accomplished using calibration lines constructed with PGE_2_ and LTB_4_ synthetic standards (Cayman Chemical, Ann Arbor, MI, USA). Data were collected and analyzed using the software Mass Lynx 4.0 (Waters).

### 2.9. Quantification of Cytokine and Chemokine Concentrations

The concentrations of IFN-*γ*, IL-6, MCP-1, and MIP-1*α* in supernatants of lung homogenates were determined using commercially available ELISA kits according to the manufacturer's instructions (R&D Systems, Minneapolis, MN, USA). Sensitivities were >10 pg/mL.

### 2.10. Analysis of Macrophage Activation by Flow Cytometry

Cells from mouse BALF samples were double-labeled for flow cytometric analysis as previously described [7], using combinations of the following fluorochrome-conjugated monoclonal antibodies: anti-F4/80-PerCP, anti-CD80-PE, anti-CD86-APC, and anti-MHC II-FITC (all from BD Bioscience, San Jose, CA, USA). Samples were analyzed in a FACSCanto flow cytometer (BD Bioscience), and the percentage of cells double-positive for F4/80^+^CD80^+^, F4/80^+^/CD86^+^, and F4/80^+^/MHC II^+^ were calculated using the FACSDiva software (BD Bioscience).

### 2.11. Apoptosis Assay

BALF cells from all groups were evaluated for apoptosis using an annexin V-FITC/propidium iodide (PI) apoptosis detection kit (BD Pharmingen), following the manufacturer's instructions. BALF cells were collected as mentioned previously, washed twice with PBS, centrifuged at 400 ×g for 15 min and suspended in annexin V binding buffer, and incubated with annexin V-FITC in total darkness for 15 min. PI was added to all samples immediately before data acquisition. A total of 10,000 events were acquired for each sample using the FACSCanto flow cytometer (Becton and Dickinson, San Jose, CA) [26]. A side-scatter gate was made to assess the apoptotic cells, and the results were evaluated by FACSDiva software and expressed as a percentage of late apoptotic cells (annexin^+^/PI^+^) from total gated cells.

### 2.12. Statistical Analysis

One-way ANOVA was used followed by Newman-Keuls multiple comparison test and Student's *t*-test to analyze differences between experimental groups. Survival differences were detected by the log-rank test. The results were considered significant when *p* < 0.05.

## 3. Results

### 3.1. EPO Increases Mortality of* Hc*-Infected Mice without Rising Fungal Burden

**Figure 1:**
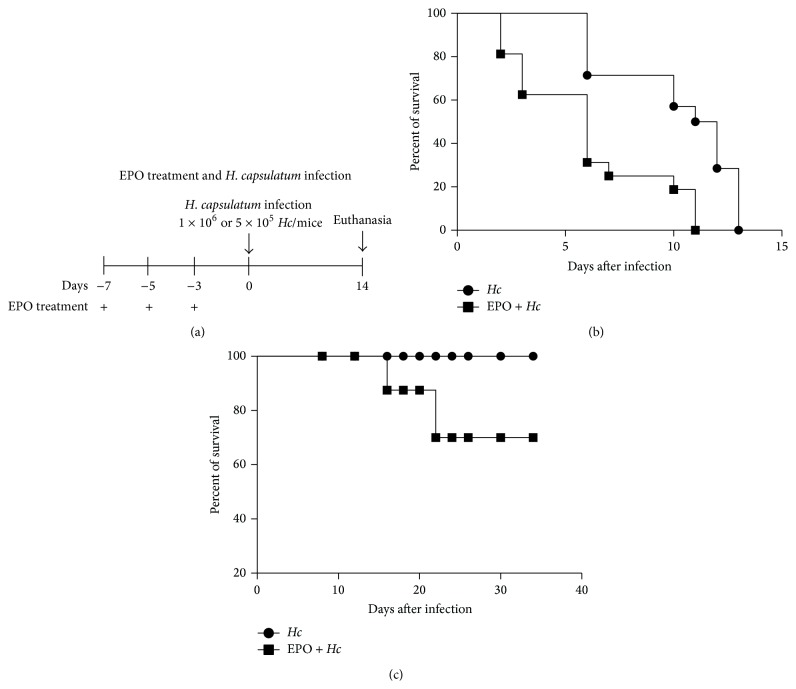
EPO pretreatment decreases the survival of* Hc*-infected mice. (a) Schematic representation of EPO treatment. In all experiments mice were treated with 45 U EPO three times per week, one week before* Hc* infection with (b) lethal (1 × 10^6^ yeasts/100 *μ*L/mouse) or (c) sublethal (5 × 10^5^ yeasts/100 *μ*L/mouse) (i.t.). Survival was followed up to 15 or 35 days, as indicated (*n* = 7). The log-rank test was used and differences were considered significant when *p* < 0.05.

**Figure 2:**
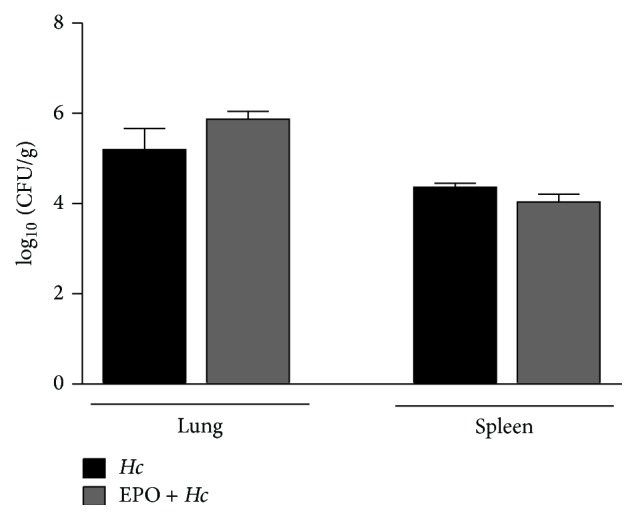
EPO pretreatment did not alter fungal burden in the lungs and spleen of* Hc*-infected mice.* Hc* and* EPO* +* Hc* animals were infected i.t. with sublethal (5 × 10^5^ yeasts/100 *μ*L/mouse) inoculum and fungal burden determined in lungs and spleen 14 days later. Fungal burden was expressed as log_10_ of the number of colony-forming units per gram of tissue (CFU/g). Mean ± SEM values are from one representative of two independent experiments (*n* = 4–6). Non differences were determined using Newman-Keuls multiple comparison test.

We observed that pretreatment with EPO (Figure 1(a)) increased the mortality of sublethal or lethal* Hc*-infected mice (Figures 1(b) and 1(c)). The survival of non infected mice was not altered by EPO treatment following the same schedule (data not shown). Next, we investigate if increased mortality of infected EPO pretreated mice was due to augmentation of fungal burden in the lungs and spleen. For this experiment, 14-day postinfection fungal burden in lung and spleen was determined by counting CFU. As seen in Figure 2, EPO pretreatment did not alter the fungal burden in the organs, when compared to that observed in infected mice without EPO treatment. These results suggest that the increased mortality observed in EPO-treated mice was not due to increased fungal proliferation or dissemination.

### 3.2. EPO Decreases the Number but Increases Apoptosis of BALF Mononuclear Cells

**Figure 3:**
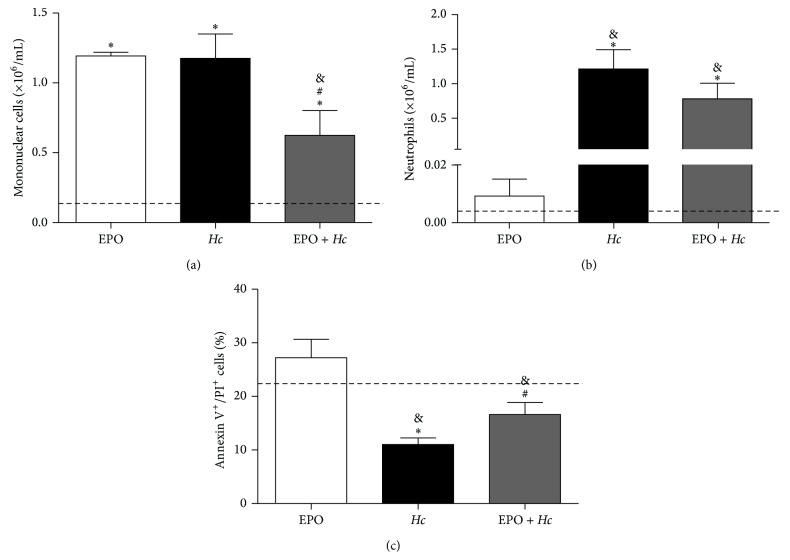
EPO pretreatment decreases the number of mononuclear cells and increases the number of cells in late apoptosis in BALF samples of* Hc*-infected mice. 14 days after infection, BALF from uninfected mice treated with 45 U of EPO (EPO) and from untreated (*Hc*) or EPO pretreated (*EPO* +* Hc*) mice infected with* Hc* (5 × 10^5^ yeasts/100 *μ*L/mouse) were evaluated for numbers of (a) mononuclear cells and (b) neutrophils. (c) Flow cytometry using annexin/PI staining was used to detect late apoptotic cells (annexin^+^/PI^+^). ((a) and (b)) Values represent means ± SEM and (c) means ± SD of one representative of two independent experiments (*n* = 4–6). Newman-Keuls multiple comparison test was used and the differences were considered significant when *p* < 0.05. ^#^Versus* Hc*; ^*∗*^versus uninfected and EPO-untreated (dashed line); ^&^versus uninfected and EPO-treated mice.

The effect of EPO pretreatment on the cell populations in the bronchoalveolar space with or without infection was investigated. We observed a decrease in mononuclear cell counts (Figure 3(a)) without a change in neutrophil numbers (Figure 3(b)) in the BALF of EPO pretreated and* Hc*-infected mice (*EPO* +* Hc*), when compared to infected EPO-untreated mice (*Hc* group). Interestingly, EPO treatment of non infected animals resulted exclusively in significant increases in mononuclear cells in the bronchoalveolar space (Figure 3(a)). Since EPO pretreatment reduced the number of mononuclear cells in the BALF of infected mice, we investigated whether this was due to increased apoptosis of mononuclear cells. The results demonstrate a decrease in the number of late apoptotic cells (annexin^+^/PI^+^) in BALF from the* Hc* group, when compared to control uninfected mice (Figure 3(b)). However, EPO pretreatment increased the number of late apoptotic cells present in BALF (*EPO* +* Hc* group), in comparison to the* Hc* group. No differences were found in the number of early apoptotic or necrotic cells between EPO pretreated mice and untreated controls (data not shown).

### 3.3. EPO Increases Alveolar Macrophage Activation, Inflammation, and Lung Damage after Fungal Infection

**Figure 4:**
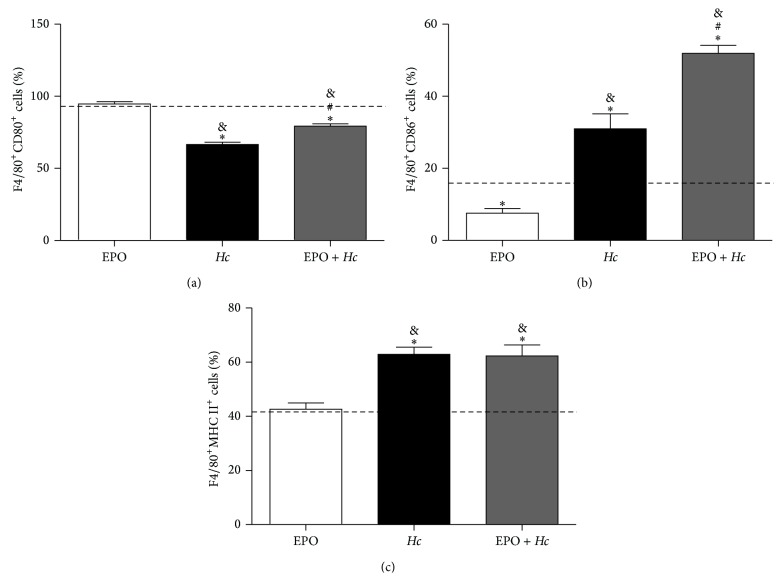
EPO pretreatment alters the expression of macrophage activation markers in* Hc*-infected mice. 14 days after infection, BALF cell populations from uninfected mice EPO-treated (EPO) and from untreated (*Hc*) or EPO pretreated (*EPO* +* Hc*) mice infected with* Hc* (5 × 10^5^ yeasts/100 *μ*L/mouse) were analyzed by flow cytometry for the percentage of F4/80^+^CD80^+^ (a), F4/80^+^CD86^+^ (b), and F4/80^+^MHC II^+^ (c) cells. Means ± SEM values from one representative of two independent experiments are shown (*n* = 4–6). Newman-Keuls multiple comparison test was used and the differences were considered significant when *p* < 0.05. ^#^Versus* Hc*; ^*∗*^versus uninfected and EPO-untreated (dashed line); ^&^versus uninfected and EPO-treated mice.

To understand why EPO treatment increased the mortality of* Hc*-infected mice without increasing fungal burden, we also investigated the degree of BALF macrophage activation and lung inflammation. Thus, the expression of the activation markers CD80, CD86, and MHC II was evaluated in F4/80^+^ cells from BALF. F4/80^+^ is a pan marker of macrophage differentiation [27]. In* EPO* +* Hc* BALF higher numbers of F4/80^+^ cells expressing CD80 and CD86 molecules were found (Figures 4(a) and 4(b)), while the proportion of F4/80^+^ cells expressing MHC II was not affected (Figure 4(c)), in comparison to only EPO-treated mice. However, the expression of CD80 molecules was decreased in* EPO* +* Hc* and* Hc* animals, when compared to uninfected control and to EPO-treated animals (Figure 4(a)).

**Figure 5:**
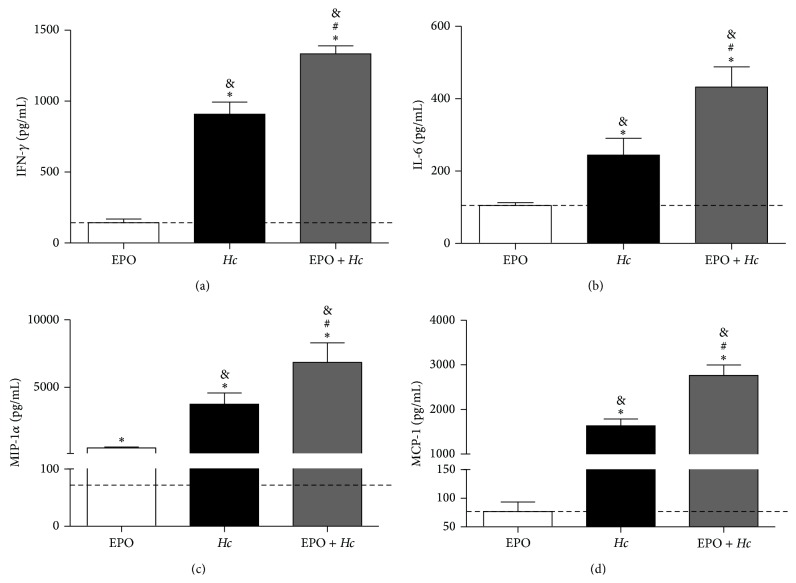
EPO pretreatment increases proinflammatory cytokines and chemokines in lungs from* Hc*-infected mice. Lungs were removed from uninfected mice treated with 45 U EPO (EPO) or from untreated (*Hc*) or EPO pretreated (*EPO* +* Hc*) mice infected with* Hc* (5 × 10^5^ yeasts/100 *μ*L/mouse). Lungs were removed 14 days after infection and homogenized, and the concentrations of IFN-*γ* (a), IL-6 (b), MIP-1*α* (c), and MCP-1 (d) were assessed in the supernatants using ELISA. Means ± SEM values from one representative experiment of two independent experiments are shown (*n* = 4–6). Newman-Keuls multiple comparison test was used and the differences were considered significant when *p* < 0.05. ^#^Versus* Hc*; ^*∗*^versus uninfected and EPO-untreated (dashed line); ^&^versus uninfected and EPO-treated mice.

**Figure 6:**
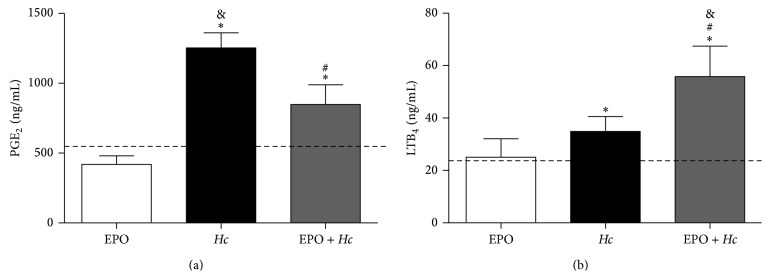
EPO pretreatment alters PGE_2_ and LTB_4_ production in lungs from* Hc*-infected mice. Lungs were removed from uninfected mice treated with 45 U EPO (EPO) or from untreated (*Hc*) or EPO pretreated (*EPO* +* Hc*) mice infected with* Hc* (5 × 10^5^ yeasts/100 *μ*L/mouse). Lungs were removed 14 days after infection and homogenized, and eicosanoids were purified as described in the Materials and Methods. PGE_2_ (a) and LTB_4_ (b) concentration was quantified in the purified homogenates by HPLC-MS/MS. Means ± SEM values from one representative experiment are shown (*n* = 4–6). Newman-Keuls multiple comparison test was used and the differences were considered significant when *p* < 0.05. ^#^Versus* Hc*; ^*∗*^versus uninfected and EPO-untreated (dashed line); ^&^versus uninfected and EPO-treated mice.

*Hc* infection increased IFN-*γ*, IL-6, MIP-1*α*, and MCP-1 production in lungs relative to those observed in uninfected and untreated mice. EPO pretreatment further increased cytokine and chemokine concentrations in infected mice (Figure 5).* Hc* infection increased TNF-*α*, KC (IL-8), and RANTES, but no further alterations were observed in the* EPO* +* Hc* group (data not shown). Because lipid mediators might contribute to lung inflammation, we evaluated LTB_4_ and PGE_2_ production in the lungs of infected mice with or without EPO pretreatment. In the* EPO* +* Hc* group, we observed decreased PGE_2_ (Figure 6(a)) but increased LTB_4_ (Figure 6(b)) concentrations compared to that found in the* Hc* group. Infected mice treated or not with EPO showed higher amounts of eicosanoids than uninfected animals, EPO-treated or not (Figure 6).

**Figure 7:**
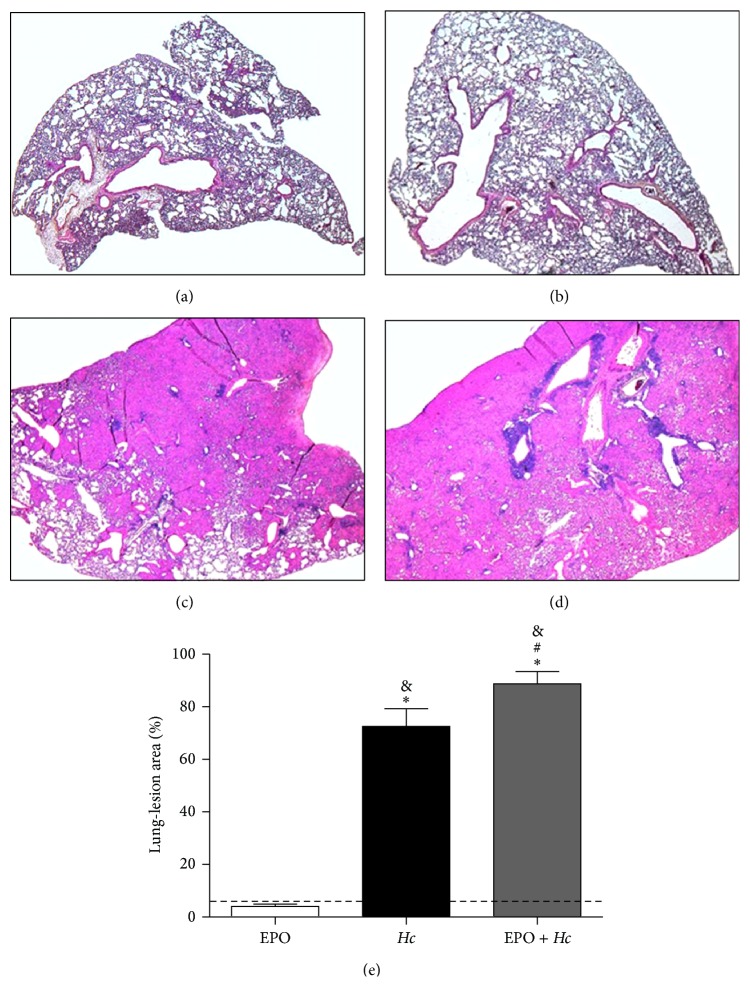
EPO pretreatment increases the area of lung tissue damage in* Hc*-infected mice. Lungs removed from uninfected and untreated mice (CTL) (a), EPO pretreated and uninfected mice (EPO) (b),* Hc-*infected only (*Hc*) (c), and EPO pretreated mice infected (5 × 10^5^ yeasts/100 *μ*L/mouse) (*EPO* +* Hc*) (d). Lungs were removed 14 days after infection, processed, and stained using H&E for routine histological examination. (e) Lung-lesion area was calculated by measuring three lungs per group and was expressed as total lung area using Adobe Photoshop. Mean ± SEM values from one independent experiment are shown. Representative photomicrography of lung of one animal per group from *n* = 4–6. Newman-Keuls multiple comparison test was used and the differences were considered significant when *p* < 0.05. ^#^Versus* Hc*; ^*∗*^versus CTL (dashed line); ^&^versus EPO.

As we observed increased inflammatory mediator concentrations, we assessed lung inflammation and parenchymal damage by histopathological analysis. Corroborating our previous data [5], we observed that* Hc* infection induced lung inflammation with severely compromised lung architecture (Figures 7(c) and 7(d)). EPO pretreatment of infected mice clearly increased lung inflammation and damage, as seen by the expansion of LLAs (Figures 7(d) and 7(e)). We observed that EPO pretreatment increased inflammatory cellular infiltration, mainly with mononuclear cells, and decreased alveolar structures in the lung parenchyma, suggesting that the impairment of respiratory capacity may be responsible for death of the mice.

Taken together, our results suggest that pretreatment with EPO decreases the survival of infected mice due to excessive lung inflammation, which severely compromises lung architecture, impairing normal respiratory capacity. Excessive inflammation in EPO pretreated mice appears to be due to increased activation of macrophages that release higher amounts of inflammatory mediators than macrophages from infected mice in the absence of EPO pretreatment.

## 4. Discussion

Nonerythroid effects of EPO have been described in several studies [11, 13, 14, 18]. Among their nonclassical effects, EPO is known as a macrophage activating factor [11] and seems to be involved in lipid mediator production [20]. Because of the importance of macrophages and lipid mediators for the control of fungal infection, the purpose of this study was to investigate the effect of EPO pretreatment in the progression of lung infection induced by* Hc*. We observed that EPO treatment for one week before* Hc* infection increased mice mortality without corresponding increases in fungal burden in the lungs or spleen, suggesting no correlation with fungal proliferation and/or dissemination. Our analysis of different aspects of the innate immune response elicited by* Hc* infection in EPO pretreated mice revealed that increased mortality may result from excessive lung inflammation due to increased macrophage activation and the production of inflammatory mediators.

Our data are in agreement with the report of Nairz et al. [19] showing that 5 U/g EPO augmented the mortality of mice infected with* S. typhimurium* as compared to infected mice in the absence of EPO. Increased mortality was accompanied with reduced NO production and cytokines involved in controlling host resistance. The production of NO is also important for the control of* Hc* infection [3, 28]. However, pretreatment with EPO did not significantly alter NO production (data not shown), and fungal burden was not increased. However, mononuclear cell numbers in the bronchoalveolar space were decreased in* EPO* +* Hc*, as compared to the* Hc* group. These results are surprising because mononuclear cells are the most efficient at triggering fungal death during* Hc* infection [3]. We also found a reduced number of apoptotic cells recovered from the BALF of* Hc*-infected mice. The pretreatment with EPO partially increased apoptosis, which might explain the reduction in mononuclear cell numbers found in BALF samples of EPO pretreated and infected mice, compared to infected mice not pretreated with EPO.

The fact that the reduction in mononuclear cell numbers in EPO pretreated mice did not result in increased fungal burden might be explained by the higher activation of alveolar macrophages observed in these mice. Pretreatment with EPO before* Hc* infection increased the frequency of CD80^+^ and CD86^+^ (but not MHC II^+^ cells) in the F4/80^+^ macrophage population from BALF samples. Similarly, Lifshitz and colleagues [11] reported that the spleens of both C57BL/6 mice treated with rHuEPO and* tg6* mice (which produce EPO excessively) had increased numbers of F4/80^+^ cells coexpressing CD80 and MHC II molecules. In a previous study using the same mouse model, these authors also showed increased frequency and expression of CD80 in CD11c^+^ cells [29]. Moreover, the pretreatment with EPO also augmented the production of inflammatory mediators such as IL-6, IFN-*γ*, MCP-1, MIP-1*α*, and LTB_4_ in* Hc* mice. Although these mediators are important for the control of infection [5, 30–33], their excessive production might be detrimental to the host by triggering excessive lung inflammation. Disproportionate lung inflammation was confirmed by histopathological analysis in the* EPO* +* Hc* group (Figure 7), which demonstrated increases in LLA. Our data are not in agreement with a previous study showing that EPO pretreatment reduced IL-6, TNF-*α*, and IL-1*β* production in the liver during* S. typhimurium* infection [19].

In addition to cytokines, the eicosanoids LT and PG also play a central role in controlling* Hc* infection [5–8]. Previous data from our laboratory demonstrated that PGs impair host control of* Hc* infection and that PG synthesis inhibition by celecoxib led to yeast elimination and infection control [8]. In the present study, we found increased PGE_2_ concentration in* Hc*-infected mice, but EPO treatment before the infection reduced PGE_2_ concentration, although no reduction in mortality was associated with this phenomenon. It is known that PGE_2_ plays a role in apoptotic events. While PGE_2_ induces apoptosis in some cell types, including resting immature and mature human lymphocytes [34] and epithelial cells [35], inhibition of apoptosis by PGE_2_ has been reported for other cell types, including neutrophils [36–38]. Similarly, in the present report, there was a negative association between PGE_2_ concentration and the number of apoptotic cells in BALF samples.

Overall, our data strongly suggest that excessive inflammation caused by exacerbated activation of alveolar macrophages increases lung inflammation, tissue damage, and mortality in EPO pretreated and infected mice. These results are consistent with a recent report from Doitsh and colleagues [39] demonstrating the role of excessive inflammation in maintaining CD4^+^ T cell death during HIV infection. This phenomenon was related to cell death of nonpermissive CD4^+^ T cells by pyroptosis, which is caused by excessive inflammation and also promotes further inflammation, maintaining the cycle of cell death and compromising the patient's quality of life.

In humans, the use of recombinant EPO is very common in patients with chronic renal disease and/or those undergoing hemodialysis, because various forms of anemia are common in these cases [40]. Anemia, which impairs quality of life, occurs primarily due to deficiency in the production of EPO by the kidney but can be exacerbated by iron deficiency, which contributes to the shortened lifespan of erythrocytes, among other complications. However, treatment is not based solely on EPO administration; antiproliferative cytokines and stimulation of iron uptake are used in combination with EPO in attempts to reverse the anemia [41].

It is important to highlight that although EPO treatment is crucial for control of anemia in patients with chronic renal disease or those undergoing hemodialysis, our study focused on inappropriate immune responses that these patients might generate prior to a potential fungal infection acquired during the treatment period, which could contribute to their morbidity.
